# Muscle-Type Nicotinic Receptor Modulation by 2,6-Dimethylaniline, a Molecule Resembling the Hydrophobic Moiety of Lidocaine

**DOI:** 10.3389/fnmol.2016.00127

**Published:** 2016-11-24

**Authors:** Armando Alberola-Die, Gregorio Fernández-Ballester, José M. González-Ros, Isabel Ivorra, Andrés Morales

**Affiliations:** ^1^División de Fisiología, Departamento de Fisiología, Genética y Microbiología, Universidad de AlicanteAlicante, Spain; ^2^Instituto de Biología Molecular y Celular, Universidad Miguel HernándezAlicante, Spain

**Keywords:** 2, 6-dimethylaniline, lidocaine, nicotinic acetylcholine receptors, *Xenopus* oocytes, microtransplanted receptors, allosteric modulation

## Abstract

To identify the molecular determinants responsible for lidocaine blockade of muscle-type nAChRs, we have studied the effects on this receptor of 2,6-dimethylaniline (DMA), which resembles lidocaine’s hydrophobic moiety. *Torpedo marmorata* nAChRs were microtransplanted to *Xenopus* oocytes and currents elicited by ACh (*I*_ACh_), either alone or co-applied with DMA, were recorded. DMA reversibly blocked *I*_ACh_ and, similarly to lidocaine, exerted a closed-channel blockade, as evidenced by the enhancement of *I*_ACh_ blockade when DMA was pre-applied before its co-application with ACh, and hastened *I*_ACh_ decay. However, there were marked differences among its mechanisms of nAChR inhibition and those mediated by either the entire lidocaine molecule or diethylamine (DEA), a small amine resembling lidocaine’s hydrophilic moiety. Thereby, the *IC*_50_ for DMA, estimated from the dose-inhibition curve, was in the millimolar range, which is one order of magnitude higher than that for either DEA or lidocaine. Besides, nAChR blockade by DMA was voltage-independent in contrast to the increase of *I*_ACh_ inhibition at negative potentials caused by the more polar lidocaine or DEA molecules. Accordingly, virtual docking assays of DMA on nAChRs showed that this molecule binds predominantly at intersubunit crevices of the transmembrane-spanning domain, but also at the extracellular domain. Furthermore, DMA interacted with residues inside the channel pore, although only in the open-channel conformation. Interestingly, co-application of ACh with DEA and DMA, at their *IC*_50_s, had additive inhibitory effects on *I*_ACh_ and the extent of blockade was similar to that predicted by the allotopic model of interaction, suggesting that DEA and DMA bind to nAChRs at different loci. These results indicate that DMA mainly mimics the low potency and non-competitive actions of lidocaine on nAChRs, as opposed to the high potency and voltage-dependent block by lidocaine, which is emulated by the hydrophilic DEA. Furthermore, it is pointed out that the hydrophobic (DMA) and hydrophilic (DEA) moieties of the lidocaine molecule act differently on nAChRs and that their separate actions taken together account for most of the inhibitory effects of the whole lidocaine molecule on nAChRs.

## Introduction

The nicotinic acetylcholine receptor (nAChR) is the prototypical member of the ligand-gated ion channel (LGIC) superfamily. This receptor mediates fast excitatory synaptic transmission in both peripheral and central nervous systems and it is essential for evoking skeletal muscle contraction (Albuquerque et al., 2009). In the past few years, a growing number of ligands have been developed to selectively modulate nAChRs, as potential tools for the treatment of different pathophysiological processes, including addiction, depression, cognitive alterations, motor dysfunctions, inflammation, and pain (Taly et al., 2009; Hurst et al., 2013; Wu et al., 2015), indicating that nAChRs constitute a chief therapeutic target.

Nicotinic acetylcholine receptor function can be modulated by a broad number of molecules, some of them containing tertiary-amino or quaternary-ammonium groups in their structure, including: (i) local anesthetics (LAs) like lidocaine (Alberola-Die et al., 2011, 2013) or its structural analogs, QX-314 and QX-222 (Neher and Steinbach, 1978; Pascual and Karlin, 1998); (ii) cholinesterase inhibitors as BW284c51, edrophonium or decamethonium (Olivera-Bravo et al., 2007) and (iii) small molecules such as choline (Grosman and Auerbach, 2000; Lape et al., 2009), TMA and TEA (Akk and Steinbach, 2003) or DEA (Alberola-Die et al., 2016). All these molecules are totally or partially protonated at physiological pH and, thus, their quaternary-ammonium group might be responsible for nAChR inhibition by open-channel blockade, acting within the channel pore (Arias, 2006). However, several LAs, as adiphenine, proadifen, or lidocaine exert multiple inhibitory actions on nAChRs (Spitzmaul et al., 2009; Alberola-Die et al., 2011, 2013), which cannot be solely explained by the interaction of an ammonium group within the channel pore, because they also enhanced desensitization and caused closed-channel blockade. For these reasons, hydrophobic aromatic rings, which are present in most LAs, are expected to play a relevant role.

**FIGURE 1:**
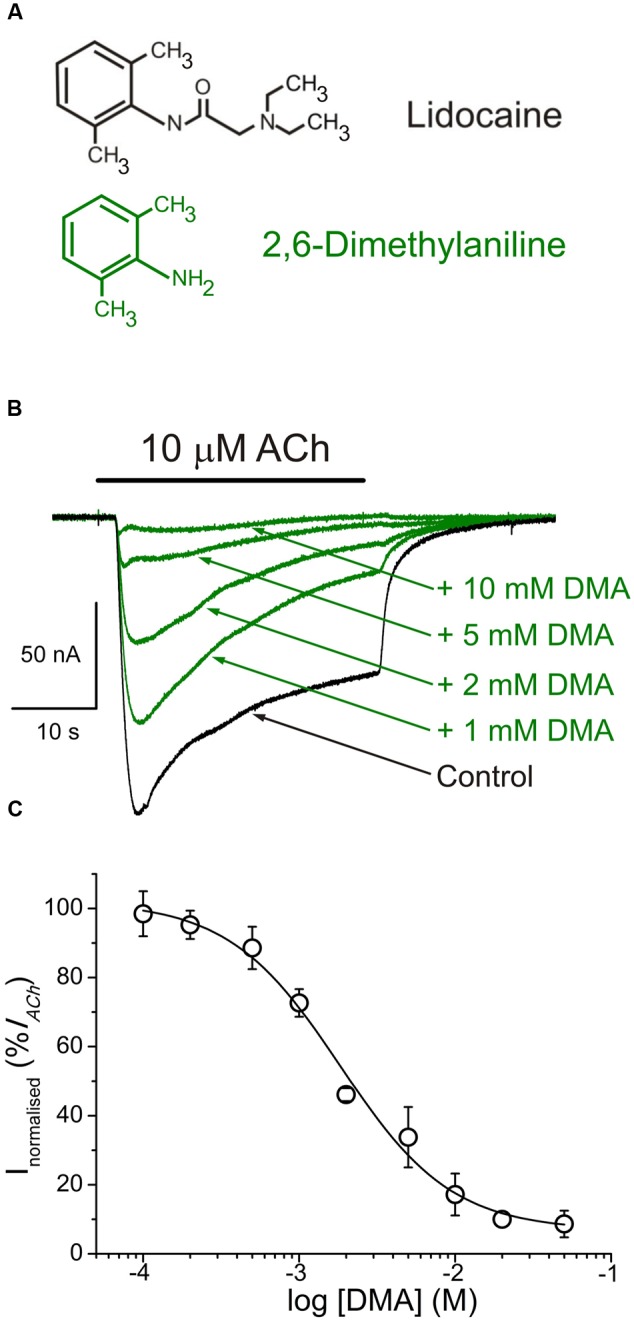
**2,6-Dimethylaniline (DMA) inhibits ACh-induced currents (*I*_ACh_s). (A)** Molecular structures of lidocaine and DMA, showing the resemblance of DMA to the phenolic ring of lidocaine. **(B)** Superimposed *I*_ACh_s, recorded in the same nAChR-bearing oocyte, by application of 10 μM ACh either alone (Control) or together with DMA, at the indicated concentrations. In this and following figures, unless otherwise stated, the holding potential was -60 mV, downward deflections denote inward currents and the horizontal bar above records corresponds to the timing of drug application. **(C)** DMA concentration-*I*_ACh_ inhibition relationship. Amplitude of the *I*_ACh_s evoked in presence of DMA was normalized to the *I*_ACh_ elicited by ACh alone (Control) and plotted as a function of the logarithm of the DMA concentration. Solid line is a sigmoid curve fitted to the data and error bars are SEM. Each point is the average of 4–28 oocytes from 3 to 13 frogs.

In a previous work we have found that DEA, a structural analogous of lidocaine’s hydrophilic moiety, mimics some, but not all, of the modulating effects of the entire lidocaine molecule on muscle-type nAChRs (Alberola-Die et al., 2016). Consequently, the present study is aimed, first, to unravel the effects of DMA, which resembles lidocaine’s hydrophobic ring (see molecular structures in **Figure 1A**), on this receptor and to decipher the nAChR loci at which DMA binds. The second goal is to correlate the mechanisms of action of DMA on nAChRs with those reported for either the entire lidocaine molecule (Alberola-Die et al., 2011) or the hydrophilic moiety of lidocaine, DEA (Alberola-Die et al., 2016). Our results indicate that although both DEA and DMA block nAChRs, their mechanisms of action and binding sites on this receptor are markedly different.

Preliminary results have previously appeared in a conference abstract (Alberola-Die et al., 2009).

## Materials and Methods

### Purification and Reconstitution of nAChRs

Nicotinic acetylcholine receptors from *Torpedo marmorata* electroplax were purified by bromoacetylcholine-affinity chromatography in the presence of asolectin lipids using cholate as a detergent. After elution with carbamylcholine, purified receptors were dialyzed and reconstituted in asolectin lipids at a final protein concentration of 0.3–1.2 mg ml^-1^. Samples were aliquoted and stored in liquid nitrogen (Ivorra et al., 2002).

### Oocyte Preparation and Microinjection

Adult female *Xenopus laevis* (purchased from Harlan Interfauna Ibérica S.L., Barcelona, Spain; and Centre National de la Recherche Scientifique, Montpellier, France) were immersed in cold 0.17% MS-222 for 20 min and a piece of ovary was drawn out aseptically. Animal handling was carried out in accordance with the guidelines for the care and use of experimental animals adopted by the E.U. and the animal protocol was approved by the Ethic Committee of Universidad de Alicante. Stage V and VI oocytes were isolated and their surrounding layers removed manually. Cells were kept at 15–16°C in a modified Barth’s solution [88 mM NaCl, 1 mM KCl, 2.40 mM NaHCO_3_, 0.33 mM Ca(NO_3_)_2_, 0.41 mM CaCl_2_, 0.82 mM MgSO_4_, 10 mM HEPES (pH 7.4), 100 U ml^-1^ penicillin and 0.1 mg ml^-1^ streptomycin] until used. Oocytes were microinjected with 100 nl of an aliquot of reconstituted nAChRs (Morales et al., 1995).

### Two-Electrode Voltage-Clamp Recordings in Oocytes

Membrane current recordings were performed at 21–25°C, 16–72 h after proteoliposome injection, using a high compliance two-microelectrode voltage-clamp system (TurboTEC-10CD, npi Tamm, Germany). The recording methodology has been described previously (Morales et al., 1995; Alberola-Die et al., 2016). Briefly, oocytes were placed in a 150 μl recording chamber and continuously superfused with normal frog Ringer’s solution (NR: 115 mM NaCl, 2 mM KCl, 1.8 mM CaCl_2_, 5 mM HEPES, pH 7.0) supplemented with 0.5 μM atropine sulfate (ANR) to block any muscarinic response (Kusano et al., 1982). The membrane potential was held at -60 mV, unless otherwise stated. ACh and other tested drugs were diluted in ANR solution and oocytes were superfused with them at a flow rate of 13–17 ml min^-1^. Membrane currents elicited by ACh (*I*_ACh_) either alone or co-applied with DMA, were low-pass filtered at 30–1000 Hz and, after sampling at fivefold the filter frequency (Digidata series 1200 and 1440A; Axon Instruments, Foster City, CA, USA), recorded on two PC-computers, using the WCP v. 3.2.8 package developed by J. Dempster (Strathclyde Electrophysiology Software, University of Strathclyde, Scotland, UK) and AxoScope v. 10.0.0.60 (Molecular Devices Corporation, Sunnyvale, CA, USA).

### Experimental Design

Experimental procedures were similar to those used to study the modulating effects of lidocaine (Alberola-Die et al., 2011) and DEA (Alberola-Die et al., 2016) on nAChRs. Briefly, DMA concentration-*I*_ACh_ inhibition relationship was determined by measuring *I*_ACh_s evoked by 10 μM ACh alone or together with different DMA concentrations. For competition assays, ACh concentration-*I*_ACh_ amplitude curves were obtained by exposing injected oocytes to increasing ACh concentrations, either alone or together with 2 mM DMA. *I*_ACh_s were recorded in the presence or absence of DMA, normalized to the *I*_ACh_ evoked by 1 mM ACh alone, and the values fitted to a sigmoid curve (see below Eq. (3)). To allow nAChRs to recover from desensitization, the interval between consecutive ACh applications was at least 5 min. To assess the blockade of resting nAChRs by DMA, we compared the *I*_ACh_s elicited by ACh (from 1 μM to 1 mM) alone or co-applied with 2 mM DMA either directly or after pre-application of DMA (same concentration) for 12 s.

The voltage dependence of the *I*_ACh_ blockade by DMA was determined by applying to the oocyte series of 800 ms voltage pulses (from -120 to +60 mV, in 20 mV steps) before ligand superfusion and during the *I*_ACh_ plateau elicited by 10 μM ACh, either alone or co-applied with DMA at different concentrations; the -120 mV pulse duration was extended up to 1500 ms to allow a more complete current relaxation.

### Data Analysis and Statistical Procedures

Inhibition curves were determined by measuring *I*_ACh_ evoked by 10 μM ACh in the presence of different DMA concentrations. *I*_ACh_s elicited in the presence of DMA were normalized to the *I*_ACh_ evoked by ACh alone. Data were fitted to a single-site inhibition curve using the Origin 6.1 software (OriginLab, Corp. Northampton, MA, USA).

Recovery from *I*_ACh_ blockade by DMA was determined by giving 32 s pulses of ACh either alone or co-applied with DMA, for solely the first 12 s or during the whole pulse; *I*_ACh_ recovery was measured 20 s and 7 min after DMA washout. The percentage of recovery from blockade (% Recovery) was obtained using the Eq. (1):

(1) $$\begin{array}{c} \%\mathrm{Recovery}=\frac{I_{\mathrm{ACh}\;\;\mathrm{after}\;\mathrm{DMA}}-I_{\mathrm{ACh}\;+\;\mathrm{DMA}}}{I_{\mathrm{ACh}}-I_{ACh+DMA}}\times 100 \end{array}$$

where *I*_ACh_ is the current amplitude evoked by 10 μM ACh alone; *I*_ACh+DMA_, is the current elicited by co-application of 10 μM ACh with 2 mM DMA; and I_AChafterDMA_ is the current obtained 20 s or 7 min after DMA removal.

The rate of desensitization (*I*_ACh_ decay) was determined by measuring the *I*_ACh_ amplitude elicited by 100 μM ACh, either alone or co-applied with different concentrations of DMA (100 μM–2 mM), at different times after *I*_ACh_ peak. Desensitization rates were obtained using the Eq. (2):

(2) $$\begin{array}{c} D_{\mathrm{ti}}=[1-(I_{\mathrm{ti}}/I_{\mathrm{peak}})]\times 100 \end{array}$$

where *D*_ti_ is the desensitization value at 2, 10, or 20 s after the peak; *I*_peak_ the *I*_ACh_ amplitude at the peak; and *I*_ti_ the current amplitudes remaining after the specified times (Olivera-Bravo et al., 2007). The apparent time-to-peak was determined as the time elapsed from *I*_ACh_ onset to the *I*_ACh_ peak, from currents elicited by ACh either alone or with DMA. We have called this parameter as “apparent” time-to-peak, just to indicate that these values do not necessarily reflect “real” time-to-peak values of nAChR activation but those observed in our experimental conditions.

To characterize the pharmacological profile of DMA, nAChRs were activated by different ACh concentrations either alone or co-applied with DMA (at roughly its *IC*_50_, unless otherwise stated), just directly or after its pre-application for 12 s. Dose-response data were fitted to the following form of the Hill Eq. (3):

(3) $$\begin{array}{c} I/I_{\max}={\left[1+{\left(EC_{50}/[\mathrm{ACh}]\right)}^{n_{H}}\right]}^{-1} \end{array}$$

where *I* is the *I*_ACh_ peak elicited at a given ACh concentration ([ACh]; applied either alone or together with DMA); *I*_max_ is the maximum *I*_ACh_ recorded; *EC*_50_ is the agonist concentration required to obtain one-half the maximum *I*_ACh_; and *n*_H_ is the Hill coefficient.

Net *i/v* curves for *I*_ACh_ were obtained by subtracting, for each voltage, the steady-state currents attained in ANR (measured during the last 100 ms of the pulse) from the corresponding ones recorded in presence of 10 μM ACh. These net *I*_ACh_ values were normalized, for each oocyte, to the ACh response at -60 mV.

To explore whether DEA and DMA molecules bind at the same loci of nAChRs, we applied both molecules, at their corresponding *IC*_50_s, together with ACh, to assess if their co-application causes additive inhibiting effects on *I*_ACh_. The extent of *I*_ACh_ inhibition was later compared to that predicted by allotopic and syntopic models (Jarvis and Thompson, 2013). The inhibition values for the allotopic model were computed with Eq. (4):

(4) $$\begin{array}{c} {\mathrm{In}}_{\mathrm{DEA},\mathrm{DMA}}\;=\;{\mathrm{In}}_{\mathrm{DEA}}{+In}_{\mathrm{DMA}}-{\mathrm{In}}_{\mathrm{DEA}}{\mathrm{In}}_{\mathrm{DMA}} \end{array}$$

where *In*_DEA,DMA_ is the predicted *I*_ACh_ inhibition when DEA and DMA are co-applied; *In*_DEA_ and *In*_DMA_ are the *I*_ACh_ inhibitions caused by DEA and DMA, respectively. The inhibition values for the syntopic model were computed with Eq. (5):

(5) $$\begin{array}{c} {\mathrm{In}}_{\mathrm{DEA},\mathrm{DMA}}=\frac{{\mathrm{In}}_{\mathrm{DEA}}+{\mathrm{In}}_{\mathrm{DMA}}-2{\mathrm{In}}_{\mathrm{DEA}}{\mathrm{In}}_{\mathrm{DMA}}}{1-{\mathrm{In}}_{\mathrm{DEA}}{\mathrm{In}}_{\mathrm{DMA}}} \end{array}$$

Unless otherwise specified, values given are the mean ± SEM; “*n”* indicates the number of oocytes and “*N”* is the number of oocyte-donor frogs from which data were obtained. When comparing two-group means of normally distributed values, the Student’s *t*-test was used; otherwise, Mann-Whitney rank-sum test was applied. Among-group differences were determined by the analysis of variance (ANOVA) and mean differences for each pair of groups were determined with the Bonferroni *t*-test. The one-sample *t*-test was used to compare the mean of an experimental group with a specified value. For the comparison of *IC*_50_ or *EC*_50_ values we used the confidence intervals computed by the curve-fitting function of Origin 6.1 software, using a percentage of confidence of 95%. The criterion of “non-overlapping 95% confidence intervals” was used to determine significant difference between *EC*_50_s. A significance level of *p* < 0.05 was considered for all cases.

### Virtual Docking Assays

Docking assays were carried out as previously described (Alberola-Die et al., 2016). Briefly, *Torpedo* nAChR structures in the closed (4 Å resolution, code 2BG9; Unwin, 2005) and open (6.2 Å resolution, code 4AQ9; Unwin and Fujiyoshi, 2012), were taken from RCSB Protein Data Bank. The edition of the protein was made using DeepView v4.1 (Guex and Peitsch, 1997) and Yasara (Krieger et al., 2002, 2004) software without further optimization. DMA, lidocaine and propofol structures (CIDs, 6896, 3676, and 4943, respectively) were taken from NCBI Pubchem database^1^. A global docking procedure was accomplished with AutoDock 4 (Morris et al., 2008) implemented in Yasara, where a total of 500 flexible docking runs were set and clustered around the putative binding sites. The program then performed a simulated annealing minimization of the complexes, which moved the structure to a nearby stable energy minimum, by using the implemented AMBER 99 force field (Duan et al., 2003). The best binding energy complex in each cluster was stored, analyzed and used to select the best orientation of the interacting partners. Figures were drawn with open source Pymol (The PyMOL Molecular Graphics System, Version 1.8 Schrödinger, LLC^2^). Yasara pH command was set to 7.0, ensuring that molecules preserve their pH dependency of bond orders and protonation patterns. In this way, DMA molecules remained during the docking procedure uncharged, but 86% of the lidocaine molecules were protonated.

### Drugs

Acetylcholine, atropine sulfate, DEA, DMA, DMSO, MS-222, penicillin and streptomycin were from Sigma (St. Louis, MO, USA). HEPES was obtained from Acros Organics (Morris County, NJ, USA). Reagents of general use were purchased from Scharlau Chemie SA (Barcelona, Spain). DMA solutions were prepared from a 1M stock solution in DMSO. All solutions were made in ANR just before each application.

## Results

### Inhibition of *I*_ACh_ by DMA

Either in uninjected cells or in oocytes bearing nAChRs, with the membrane potential held at -60 mV, DMA application did not appreciably modify the cell membrane conductance at concentrations lower than 5 mM, indicating both a lack of unspecific effect of DMA on native ion channels opened at rest and that DMA did not act as a partial agonist of nAChRs. Nevertheless, at higher DMA concentrations some oocytes showed a slight decrease in their membrane conductance (not shown), although we have not pursued the basis of this effect.

In oocytes that had incorporated nAChRs, co-application of 10 μM ACh with DMA, at concentrations ranging from 100 μM to 50 mM, inhibited peak *I*_ACh_ amplitude in a concentration-dependent manner (**Figure 1**). The half-inhibitory DMA concentration (*IC*_50_), obtained by fitting the data to the Hill equation, was 2.1 mM (confidence interval, 1.7–2.7 mM), and the Hill coefficient (*n*_H_) 1.2 ± 0.2 (**Figure 1C**), indicating that a single DMA molecule is sufficient to block the nAChR.

**FIGURE 2:**
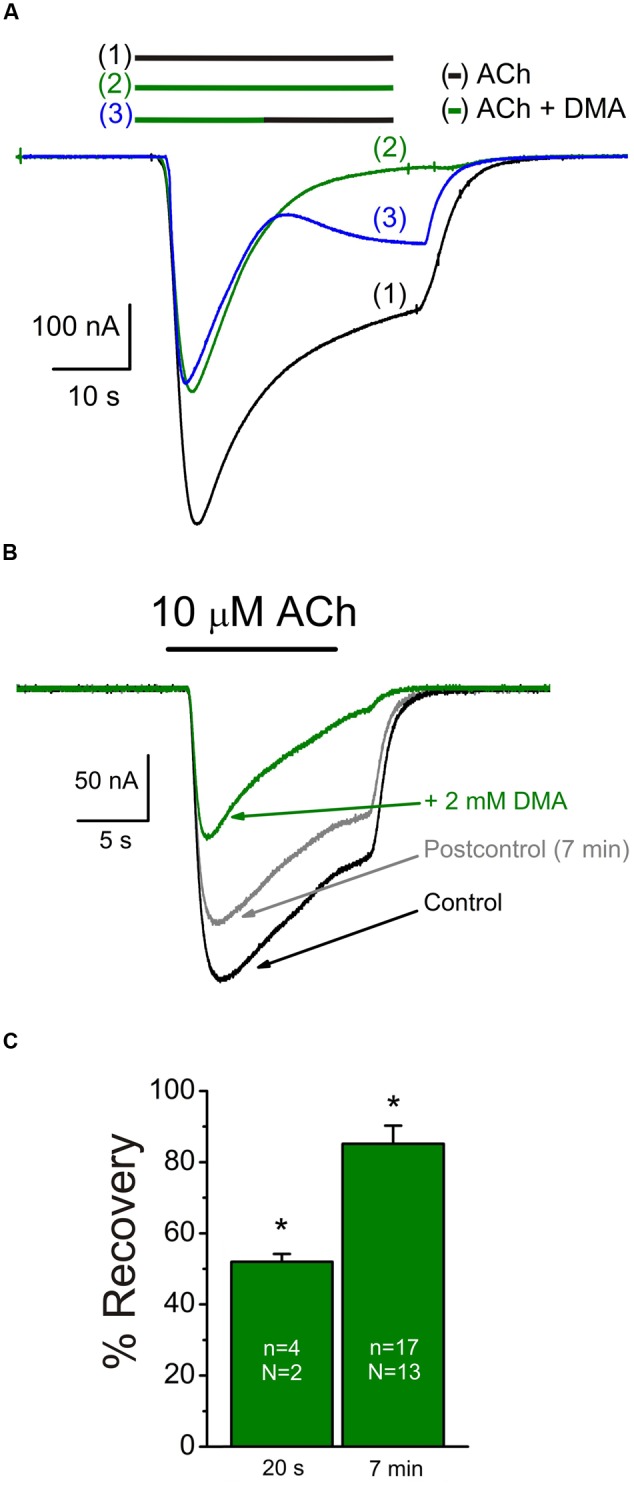
**Slow recovery from nAChR blockade by DMA. (A)** Superimposed *I*_ACh_s evoked sequentially, in the same oocyte, by superfusing the cell with 10 μM ACh alone [(1), black bar and recording], co-applied with 2 mM DMA [(2), green bar and recording] or when changing from ACh plus DMA to ACh alone at the time indicated by the bars [(3), green followed by black bars and blue recording]. Note the incomplete recovery of *I*_ACh_ amplitude after washing DMA for 20 s. **(B)** Superimposed currents obtained by superfusing one oocyte with 10 μM ACh alone (Control, black recording) or plus DMA (+ 2 mM DMA, green recording). Seven min after DMA withdrawal (Postcontrol, gray recording), *I*_ACh_ did not fully reach the control amplitude. **(C)** Column graph showing the percentages of *I*_ACh_ recovery after 20 s or 7 min from DMA washout. Asterisks indicate significant differences respect to the control response.

Nicotinic acetylcholine receptor blockade by DMA outlasted the drug application, as also occurred for lidocaine and DEA (Alberola-Die et al., 2011, 2016). Thereby, 20 s after rinsing out DMA (2 mM), the percentage of *I*_ACh_ recovery (see Materials and Methods, Eq. (1)) was only 52.0 ± 2.2% (**Figures 2A,C**), increasing to 85.1 ± 5.1% when the elapsed time was 7 min (**Figures 2B,C**). Thus, the *I*_ACh_s after both DMA washout times were significantly smaller than in control conditions, indicating a slow nAChR recovery from blockade.

### Open-Channel Blockade of nAChRs by DMA

**FIGURE 3:**
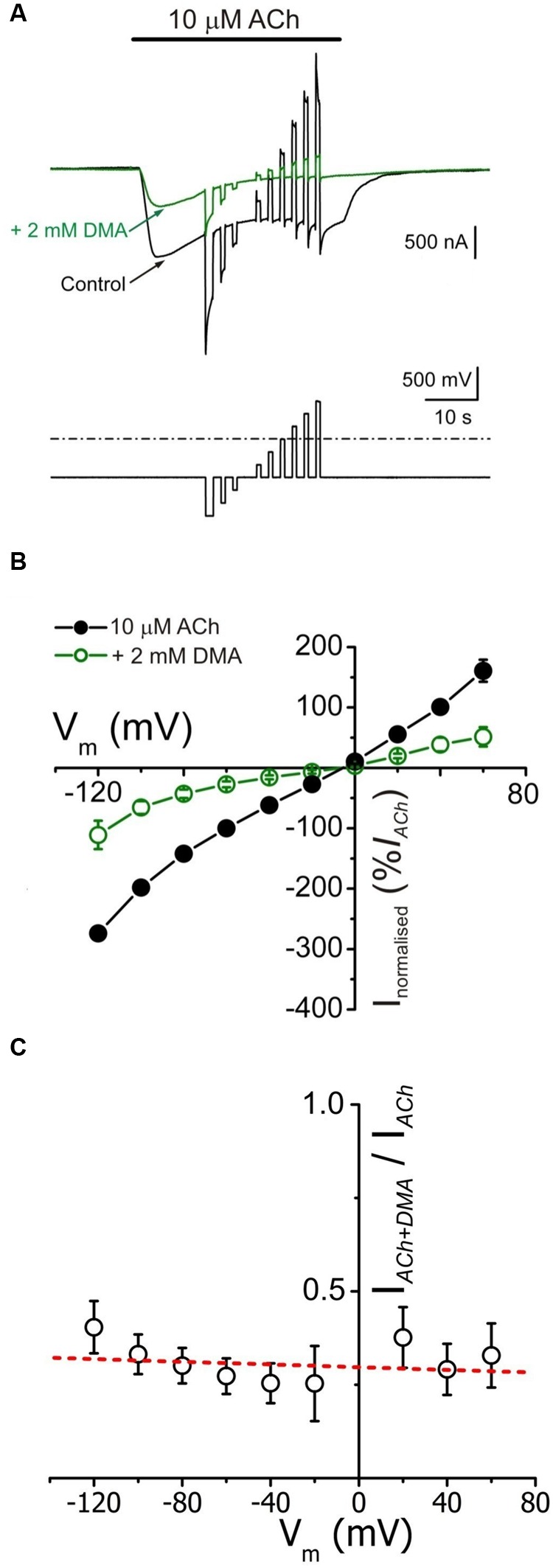
***I*_ACh_ blockade by DMA lacks of voltage dependence. (A)** Whole membrane currents (upper traces) evoked by applying to an oocyte the voltage protocol shown on bottom, during the current plateau elicited by 10 μM ACh, either alone (black) or with 2 mM DMA (green). **(B)** Net *i/v* relationships for *I*_ACh_, obtained by applying the voltage protocol shown in **(A)** while superfusing the cells with 10 μM ACh either alone (black filled circles) or co-applied with 2 mM DMA (green open circles). Values represent the percentage of current referred to their control *I*_ACh_ at -60 mV; each point is the average of 5 cells (*N* = 3). **(C)** Plot showing the fraction of plateau *I*_ACh_ left by 2 mM DMA (*I*_ACh+DMA_), normalized to its control (*I*_ACh_), versus the membrane potential. Same cells than in **(B)**. Note the lack of a clear voltage dependence of *I*_ACh_ blockade by DMA. The dashed red line shows the best linear fit to the data; the fitted line has a correlation coefficient of -0.21, giving a *p* of 0.58 (the probability for the *t*-test of the slope = 0).

We measured *I*_ACh_s at different membrane potentials by applying voltage jumps (from -120 to +60 mV, in 20 mV steps) in absence (not shown) or presence of 10 μM ACh applied either alone or together with 2 mM DMA (**Figure 3A**) to determine if *I*_ACh_ inhibition by DMA has any voltage-dependence, which would suggest its binding into the channel pore.

The *i/v* curves of net *I*_ACh_s (see Materials and Methods) elicited by ACh either alone or co-applied with 2 mM DMA are shown in **Figure 3B**. The presence of DMA did not affect the *I*_ACh_ reversal potential, thus the channel ion selectivity was unaffected. However, 2 mM DMA reduced *I*_ACh_ amplitude in a similar percentage at all tested potentials (**Figures 3A,B**), indicating that DMA caused a voltage-independent blockade of nAChRs. This lack of voltage-dependence of *I*_ACh_ blockade by DMA is more evident when plotting the percentage of *I*_ACh_ remaining after co-applying ACh with DMA, normalized to its control *I*_ACh_, against membrane potential (**Figure 3C**). Notice that when measured at the *I*_ACh_ plateau, the current left upon 2 mM DMA was roughly 30% of the control values at any potential tested (**Figure 3C**). This percentage of *I*_ACh_ remaining is fairly smaller than that found when considering the *I*_ACh_ peak (40–50%, **Figure 1C**) and this discrepancy is most likely due to the enhancement of *I*_ACh_ decay by DMA (see below).

**FIGURE 4:**
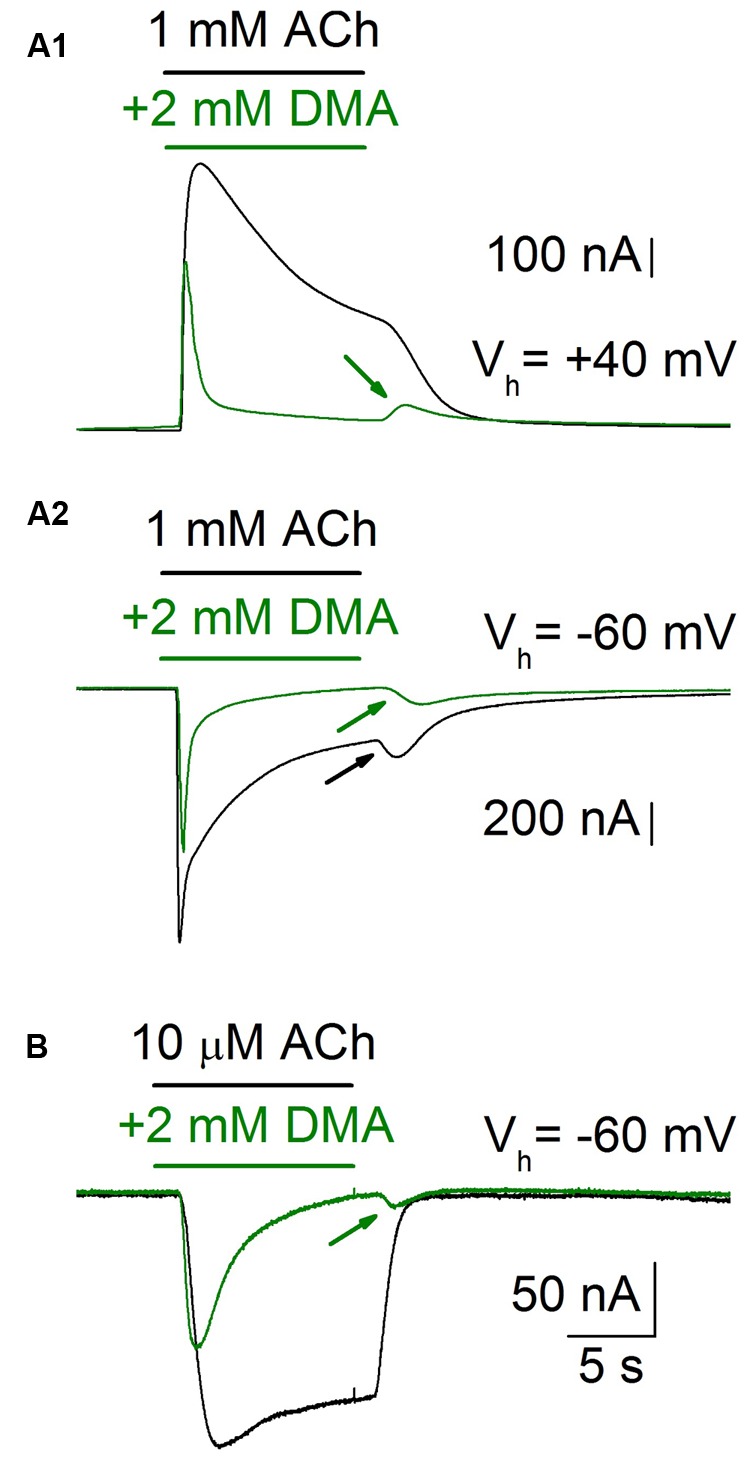
***I*_ACh_ rebound elicited by DMA washout.** When an oocyte was challenged with a high ACh concentration (1 mM), while holding its membrane potential at -60 mV (V_h_ = -60 mV), the *I*_ACh_ showed a marked desensitization and a noticeable rebound-current (**A_2_**, black recording and arrow) when the agonist was rinsed. By contrast, both when applying the same ACh concentration to the cell at a membrane potential of +40 mV (**A_1_**, black recording), or when decreasing the ACh concentration to 10 μM (**B**, black recording), the *I*_ACh_ rebound was not evoked. However, when ACh was co-applied with 2 mM DMA the *I*_ACh_ rebound was evident at any potential or ACh concentration tested (**A_1_**,**A_2,_B**, green recordings and arrows).

Since DMA is a non-charged molecule, the lack of voltage-dependence of nAChRs blockade by DMA does not fully exclude that this molecule can bind into the channel pore. Therefore, to ascertain if DMA actually binds into the channel pore we analyzed the “rebound” currents elicited by ACh either alone or in the presence of 2 mM DMA (**Figure 4**). It is well-established that high doses of ACh elicit open-channel blockade of nAChRs, evidenced by an *I*_ACh_ rebound just when rinsing out the agonist. This current arises during the agonist washout because then the ACh leaves the channel, unplugging the pore, when it can be still bound to the high affinity orthosteric sites (Legendre et al., 2000; Liu et al., 2008). This open-channel blockade of nAChRs by high ACh concentrations is only found at negative membrane potentials, because at positive voltages the positively charged ACh is electrostatically repelled from the channel pore (compare control *I*_ACh_s, black recordings, of **Figures 4A_1_,A_2_**). However, when 1 mM ACh was co-applied with 2 mM DMA, rebound currents were elicited both at positive and negative potentials (**Figures 4A_1_,A_2_**, green traces), indicating that the uncharged DMA is binding into the channel pore with low affinity, and thereby eliciting an open-channel blockade of nAChRs. Furthermore, this *I*_ACh_ rebound was also elicited when 2 mM DMA was co-applied with a low ACh concentration (10 μM) at negative potentials, in spite of the fact that ACh, at this concentration, cannot block by its own the channel pore (**Figure 4B**). Noticeably, when DMA concentration decreased below 500 μM, this rebound currents were not elicited (see in the recordings of **Figure 1B** that the rebound current appears at 2 mM DMA) and they were of larger amplitude when the cell was challenged with a relatively high ACh concentration (100 μM or higher; compare recordings of panels A_2_,B of **Figure 4**).

### DMA Enhanced *I*_ACh_ Decay and Decreased the Time-to-Peak

**FIGURE 5:**
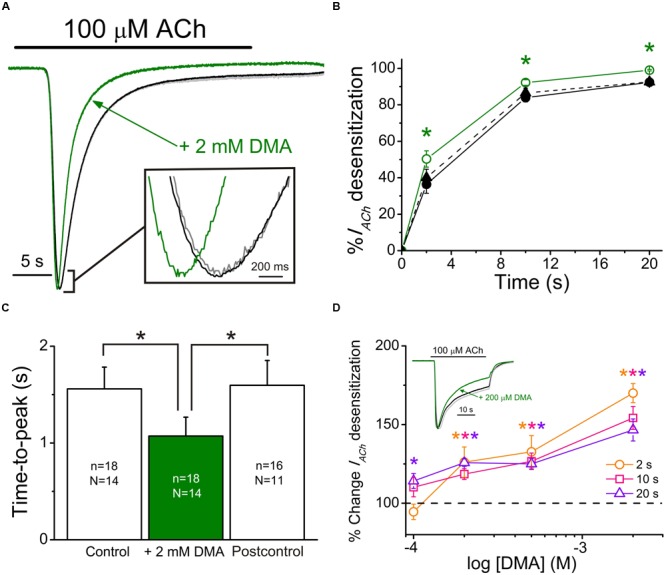
**2,6-Dimethylaniline effects on *I*_ACh_ decay and time-to-peak. (A)** Superimposed *I*_ACh_ recordings evoked by application of 100 μM ACh either alone (black recoding) or plus 2 mM DMA (green recording) and by re-applying 100 μM ACh alone 7 min after DMA washout (Postcontrol, gray trace overlapping the control one). Note that all *I*_ACh_ amplitudes have been scaled to the same size to better showing differences on *I*_ACh_ desensitization. Inset shows, at an expanded temporal scale, the *I*_ACh_ peaks elicited by ACh either alone or co-applied with DMA. **(B)** Plots showing the percentage of *I*_ACh_ decay obtained at different times (2, 10, and 20 s) after *I*_ACh_ peak. Data were measured from recordings as those shown in **(A)**, by applying 100 μM ACh either alone (Control, filled circles and continuous black line; Postcontrol, filled triangles and dashed black line) or plus 2 mM DMA (open circles and continuous green line). **(C)** Column graph showing the *I*_ACh_ time-to-peak values when applying 100 μM ACh either alone (Control and Postcontrol, empty columns) or together with 2 mM DMA (filled green column). Values of *n* and *N*, given in each column, are common to **(B,C)**; in both panels, asterisks indicate significant differences among groups (*p* < 0.05, ANOVA and Bonferroni *t*-test). **(D)** Plot displays the DMA dose-dependence of *I*_ACh_ decay hastening. Desensitization values (D_ti_s) at 2 (orange), 10 (pink) and 20 s (violet) from *I*_ACh_ peaks, elicited by co-applying 100 μM ACh with 100, 200, 500, or 2000 μM DMA, were expressed as percentage respect to their control D_ti_s and plotted against the log of DMA concentration. Each point is the average of 4–12 oocytes from three frogs. Asterisks of different colors indicate significant differences respect to the control values for the color-coded time (*p* < 0.05, one sample *t*-test). Inset shows superimposed recordings evoked by 100 μM ACh either alone or together with 200 μM DMA; recording colors are as in **(A)** and *I*_ACh_ amplitudes have also been scaled to the same size.

When either 10 or 100 μM ACh were co-applied with DMA, at roughly its *IC*_50_, *I*_ACh_ decays were significantly accelerated with respect to those evoked by ACh alone, suggesting an enhancement of nAChR desensitization by DMA. *D*_ti_ values at 2 and 20 s (see Eq. (2) in Materials and Methods) were: 36 ± 5% and 92 ± 1%, for 100 μM ACh alone versus 50 ± 5% and 99 ± 1% for 100 μM ACh plus 2 mM DMA, respectively (same cells in both groups; *n =* 18, *N* = 14; *p* < 0.05, ANOVA; see **Figures 5A,B**). This effect was fully reverted 7 min after DMA rinsing with ANR (40 ± 5% and 93 ± 2%; *p* > 0.05, ANOVA, **Figure 5B**). Additionally, DMA diminished the apparent time-to-peak, i.e., the time elapsed from *I*_ACh_ onset to *I*_ACh_ peak, from 1.6 ± 0.2 s for 100 μM ACh alone to 1.1 ± 0.2 s for 100 μM ACh plus 2 mM DMA (same cells that *I*_ACh_ decay measurements; *p* < 0.05, ANOVA; **Figures 5A,C**). Noteworthy, the time-to-peak reverted to control values 7 min after DMA washout (1.6 ± 0.3 s; see Postcontrol of **Figures 5A,C**), as the *I*_ACh_ decay rate did. Interestingly, DMA hastening of *I*_ACh_ decay was dose-dependent, starting the increase of desensitization at concentrations as low as 100 μM DMA (**Figure 5D**; *p* < 0.05, one sample *t*-test).

**FIGURE 6:**
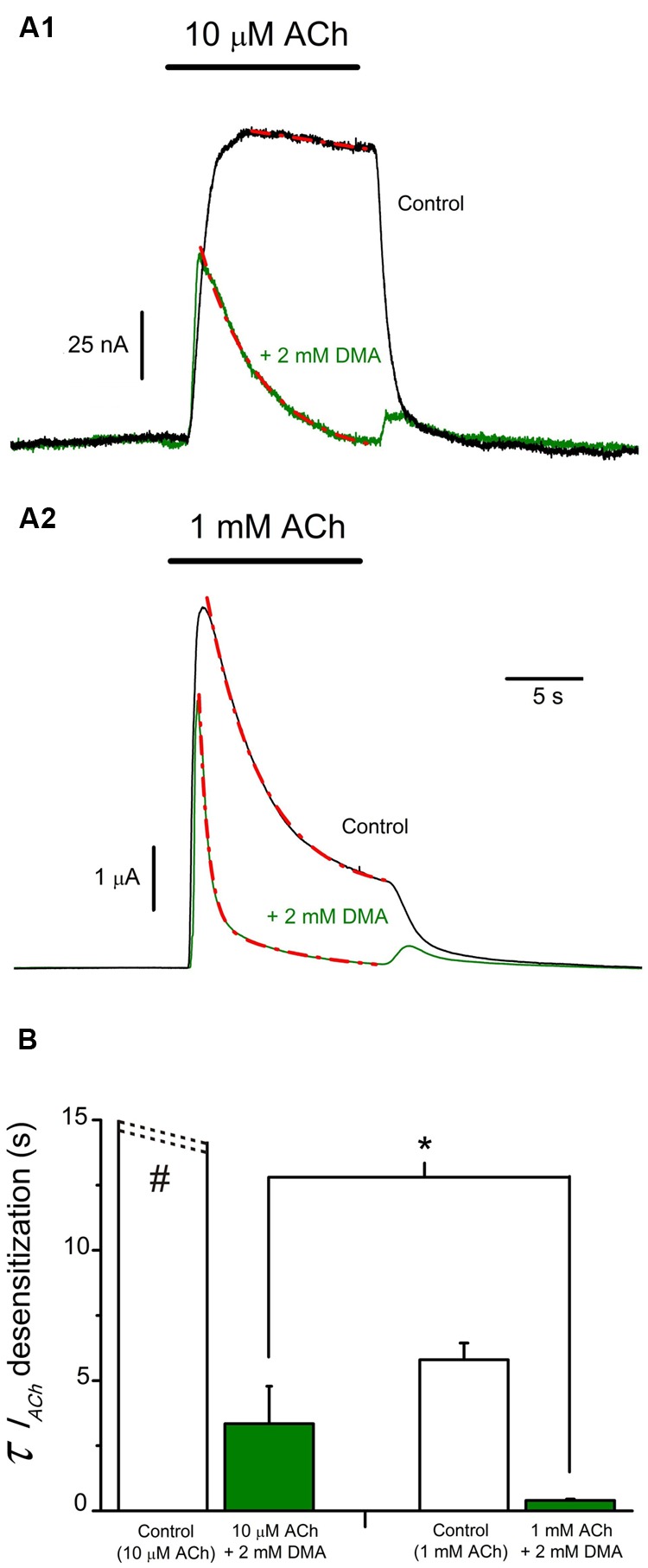
***I*_ACh_ decay hastening elicited by DMA is dependent on ACh concentration. (A)** Superimposed recordings of *I*_ACh_s elicited by 10 μM **(A_1_)** or 1 mM **(A_2_)** ACh either alone (black recordings) or co-applied with 2 mM DMA (green recordings) in oocytes with the membrane potential held at +40 mV. *I*_ACh_ decays were fitted to exponential curves (red discontinuous lines) and the time constant (τ) values for each group were determined. **(B)**. Column graph of τ values for *I*_ACh_ decays. Data of each column are mean ± SEM from 4 to 6 oocytes (*N* = 3). When co-applying DMA and 1 mM ACh, the *I*_ACh_ decay was best fitted to double exponential curves and the τ value shown in **B** corresponds to the fast component. Note that both control *I*_ACh_ amplitude and desensitization rate increased with ACh concentration (see black recordings in **A_1,_A_2_**; **B**, open columns) and mind the presence of rebound-currents when ACh and DMA were co-applied (green records), independently of the ACh dose used. Observe that DMA co-application caused a stronger blocking effect at low **(A_1_)** than at high **(A_2_)** ACh concentrations and that DMA enhancement of the rate of *I*_ACh_ decay was greater for higher ACh doses (compare recordings of **A_1_**, **A_2_**; **B**). The asterisk indicates significant differences between both DMA groups (*p* < 0,05, *t*-test), and the pound sign denotes that this column is truncated, because *I*_ACh_s elicited by 10 μM ACh showed almost no desensitization.

Since co-application of ACh with 2 mM DMA elicits rebound-currents during the washout, suggesting that DMA can plug the channel pore, the acceleration of *I*_ACh_ decay could be due either to an enhancement of nAChR desensitization or to a slow binding of DMA to the channel pore. In order to differentiate between both mechanisms, we co-applied 2 mM DMA with two different concentrations of ACh (10 μM and 1 mM) at +40 mV (**Figures 6A_1_,A_2_**), because at this membrane potential ACh does not contribute to the open-channel blockade. Co-application of 10 μM ACh with 2 mM DMA inhibited *I*_ACh_ by 50.0 ± 9.3% (*n* = 4; *N* = 3), as it would be expected from the dose-inhibition curve (**Figure 1C**), and there was a pronounced acceleration of *I*_ACh_ decay, which followed a single exponential function (**Figure 6A_1_**, red discontinuous line), with a time-constant (τ) of 3.34 ± 1.44 s (**Figure 6B**). When the same concentration of DMA was co-applied with 1 mM ACh, the *I*_ACh_ decreased by only 35.4 ± 4.3% (see **Figures 6A_2_** and **7A_1_**) and the *I*_ACh_ decayed following a double exponential function (**Figure 6A_2_**, red discontinuous line), with a τ value for the fast component of 0.40 ± 0.05 s (**Figure 6B**). Given the large differences in the τ values for the *I*_ACh_ decay caused by the same DMA concentration when co-applied with two different ACh concentrations, it follows that the acceleration of *I*_ACh_ decay by DMA cannot only be explained by its binding into the channel pore, acting as an open-channel blocker, but rather it points out that DMA actually enhances nAChR desensitization.

**FIGURE 7:**
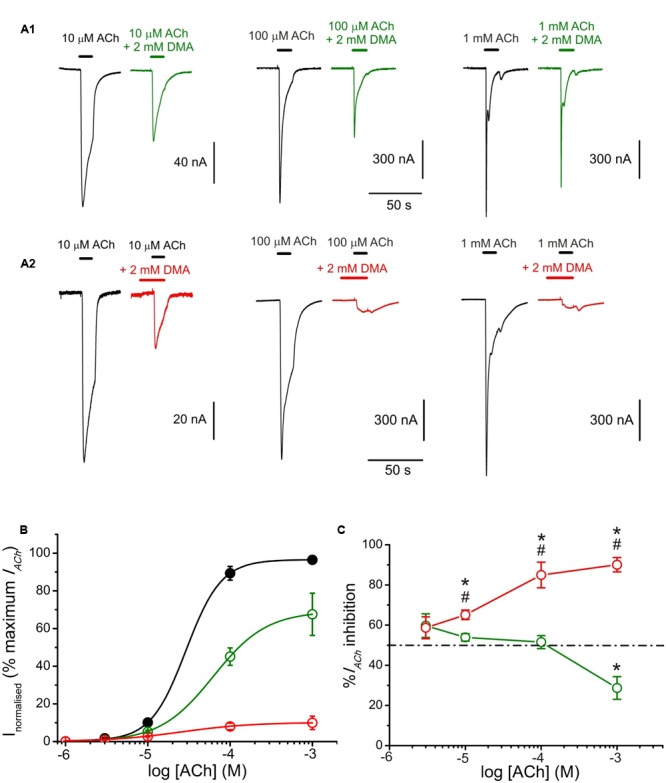
**2,6-Dimethylaniline effects on ACh concentration-*I*_ACh_ amplitude relationship. (A)** *I*_ACh_ recordings evoked by applying, successively, ACh at increasing concentrations (10, 100 μM, and 1 mM) either alone (black traces) or co-applied with 2 mM DMA either directly (**A_1_**, green recordings) or after being pre-applied for 12 s (**A_2_,** red recordings). **(B)** Averaged ACh concentration-*I*_ACh_ amplitude curves obtained following the experimental protocol shown in **(A)**. Black filled circles are for ACh alone (*n* = 10–23, *N* = 4–5), green open circles for co-application of ACh plus 2 mM DMA (*n* = 4–5, *N* = 3) and red open circles when ACh and DMA co-application was preceded by 12 s of 2 mM DMA pre-application (*n* = 4–7, *N* = 2–4). All data were normalized to the maximal *I*_ACh_ elicited by ACh alone and fitted to the Hill equation (continuous lines). **(C)** Plot showing the percentage of *I*_ACh_ inhibition at different ACh concentrations when ACh was directly co-applied with 2 mM DMA (open green circles and solid line; *n* = 4–28, *N* = 4–10), or when ACh and 2 mM DMA co-application was preceded by 12 s DMA pre-application (open red circles; *n* = 5–15, *N* = 4–9). Asterisks indicate significant differences (*p* < 0.05, ANOVA and Bonferroni *t*-test) respect to the *I*_ACh_ blockade caused by solely co-applying 10 μM ACh and 2 mM DMA; pound signs indicate significant differences (*t*-test), for each ACh dose, between the *I*_ACh_ blockade caused by direct co-application of ACh with DMA and when it was preceded by a 12 s DMA application. The dashed line indicates 50% inhibition. Note the reduction of *I*_ACh_ inhibition when DMA was co-applied with high ACh concentrations (1 mM) and the strong *I*_ACh_ blockade when DMA was pre-applied before its co-application with high ACh concentrations.

### Effects of DMA on nAChR Pharmacological Profile

The pharmacological profile of nAChR inhibition by DMA was studied by superfusing ACh at different concentrations (1, 3, 10, 100, and 1000 μM) alone or co-applied with 2 mM DMA either directly (**Figure 7A_1_**) or after 12 s pre-application of the same DMA concentration (**Figure 7A_2_**). **Figure 7B** shows the relationship between ACh-concentration and *I*_ACh_ amplitude in absence and presence of DMA. The sigmoid curve fitted for *I*_ACh_s elicited by ACh alone gave an *EC*_50_ of 29 μM (confidence interval, 22–44 μM) and an *n*_H_ of 2.0 ± 0. 1, which are similar values to those previously reported for this receptor (Morales et al., 1995; Alberola-Die et al., 2016). When 2 mM DMA was co-applied with the different ACh concentrations, the *I*_ACh_ amplitude decreased, even with the highest (almost saturating) ACh concentration (**Figures 7A_1_,B**), suggesting a non-competitive blockade of nAChRs by DMA. Furthermore, the dose-response curve shifted to the right (**Figure 7B**), increasing significantly the *EC*_50_ up to 63 μM (confidence interval, 50–96 μM) and decreasing the slope to 1.3 ± 0.1. A similar reduction in the slope of the ACh concentration-*I*_ACh_ amplitude relationship was found when co-applying ACh with the quaternary-ammonium BW284c51 (Olivera-Bravo et al., 2005) or lidocaine (Alberola-Die et al., 2011), though the mechanisms underlying this effect remains unclear, since it is only partially dependent on the increase of nAChR desensitization caused by these drugs (Alberola-Die et al., 2011). Nonetheless, given that the percentage of *I*_ACh_ inhibition was also dependent on ACh concentration, nAChR blockade by DMA was not exclusively a non-competitive antagonism. Thus, at low (10 μM) ACh concentration, 2 mM DMA blocked roughly half the control *I*_ACh_ (**Figures 7A_1_,B,C**), as expected from its estimated *IC*_50_ (see **Figure 1**). However, at very high ACh concentration (1 mM), the percentage of *I*_ACh_ blockade decreased significantly (**Figures 7A_1_,B,C**; *p* < 0.05, ANOVA). Although, we cannot fully discard some competitive interactions of DMA and ACh on the orthosteric binding sites (see below), this apparent competitive mechanism of blockade could be explained by the binding of DMA to the nAChR in its closed state, in a similar way as we have previously proposed for lidocaine and also for DEA (Alberola-Die et al., 2011, 2016). To test this hypothesis, we determined the percentages of *I*_ACh_ blockade induced by DMA (2 mM) when it was pre-applied to the oocyte for 12 s before being co-applied with ACh at increasing concentrations (1 μM–1 mM; **Figures 7A_2_,B,C**). As shown in panels 7A_2,_B, the percentage of *I*_ACh_ remaining when ACh was co-applied with 2 mM DMA, after being pre-applied for 12 s, was significantly reduced with 10 μM or higher ACh concentrations, as compared with those corresponding to solely ACh and DMA co-application. The sigmoid curve fitting the *I*_ACh_ values obtained at the different concentrations tested had an *EC*_50_ of 25 μM (confidence interval, 8–78 μM) and an *n*_H_ of 1.0 ± 0.3. Thus, pre-application of DMA followed by its co-application with 10 μM ACh increased only modestly the percentage of *I*_ACh_ inhibition obtained by barely DMA and ACh co-application (53.9 ± 1.9%, *n* = 28, *N* = 13, for direct co-application, versus 65.1 ± 2.4%, *n* = 15, *N* = 8, for pre-application followed by co-application; *p* < 0.05, *t*-test; **Figures 7A_1_,A_2_,C**). Noteworthy, the enhancement of *I*_ACh_ inhibition by DMA pre-application was stronger when it was later co-applied with high ACh concentrations, as it would be expected if DMA pre-application blocked resting nAChRs. Thereby, the percentage of *I*_ACh_ inhibition by 2 mM DMA and 1 mM ACh co-application was 28.7 ± 5.7% (*n* = 4, *N* = 2) and increased to 90.1 ± 3.5% (*n* = 5, *N* = 3; *p* < 0.05, *t*-test) when the same DMA concentration was pre-applied and then co-applied with 1 mM ACh (compare recordings of panels **Figures 7A_1,_A_2,_C**).

### Additive Inhibitory Effects of DMA with DEA

**FIGURE 8:**
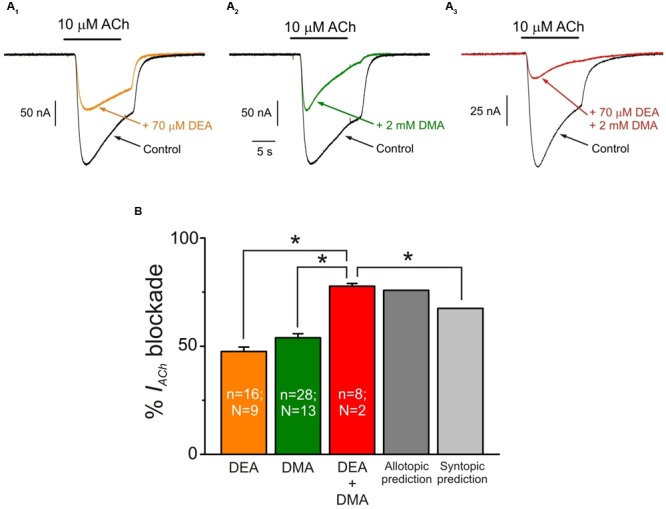
**Additive inhibitory effects of DMA and DEA on *I*_ACh_. (A_1_**–**A_3_)** Representative *I*_ACh_ recordings obtained when superfusing the oocyte with 10 μM ACh either alone (**A_1_**–**A_3_**; Control, black) or co-applied with 70 μM DEA (**A_1_**; + 70 μM DEA, orange), 2 mM DMA (**A_2_**; + 2 mM DMA, green) or 70 μM DEA plus 2 mM DMA (**A_3_**; + 70 μM DEA + 2 mM DMA, red). **(B)** Column graph showing the average *I*_ACh_ inhibition elicited by co-application of 10 μM ACh with the different combinations showed in **(A)** as indicated below each column. The two right most columns show the values predicted by the allotopic and syntopic models of interaction (see text for details). The asterisks above the bars indicate significant differences between groups (*p* < 0.05, *t*-test; comparisons of DEA + DMA values with those estimated by each model of inhibition were carried out with one-sample *t*-test).

Since DEA and DMA are structurally quite different molecules and both cause inhibitory effects on nAChRs (see Alberola-Die et al., 2016), we assessed the effect of co-application of DMA with DEA in the presence of 10 μM ACh, aiming to unravel whether or not their inhibitory actions on nAChRs are additive. Co-application of 10 μM ACh with 70 μM DEA (close to its *IC*_50_) decreased *I*_ACh_ by 47.6 ± 2.0% (**Figures 8A_1_,B**). A similar percentage of *I*_ACh_ blockade was obtained when ACh (10 μM) was co-applied with 2 mM DMA (53.9 ± 1.9%; **Figures 8A_2_,B**). The *I*_ACh_ inhibition increased significantly when these same doses of DMA and DEA were co-applied together with 10 μM ACh (77.7 ± 1.3%; **Figures 8A_3_,B**; *p* < 0.05, *t*-test). This enhancement of *I*_ACh_ inhibition by co-application of DEA and DMA could be due to either syntopic (both molecules sharing a single binding site) or allotopic (binding to different loci) interaction of these molecules on the nAChR. Using the theoretical approach proposed by Jarvis and Thompson (2013) to discriminate between both interaction models (see Materials and Methods Eqs. (4) and (5)), we found that *I*_ACh_ inhibition caused by DEA and DMA co-application properly fitted to the values predicted by the allotopic model, but were significantly different to those estimated by the syntopic one (**Figure 8B**).

**FIGURE 9:**
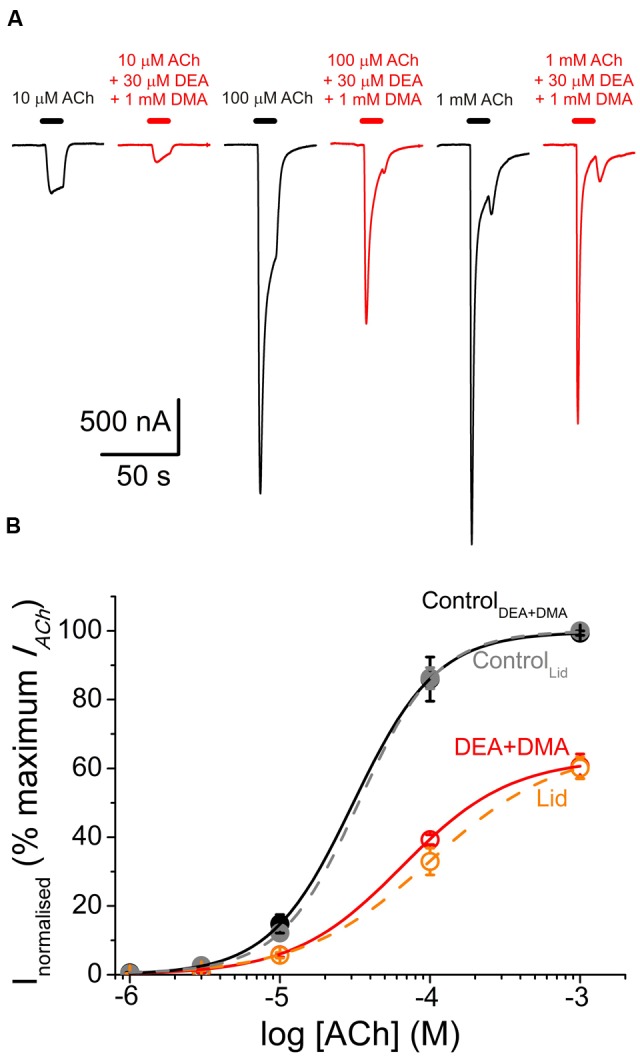
**Similar pharmacological profile of nAChRs in the presence of either combined DEA and DMA or lidocaine. (A)** *I*_ACh_ recordings elicited by applying, sequentially, ACh at increasing concentrations (10, 100 μM, and 1 mM) either alone (black traces) or co-applied with 1 mM DMA and 30 μM DEA (red traces). **(B)** Averaged ACh concentration-*I*_ACh_ amplitude curves attained following the experimental protocol shown in **(A)**. Black filled circles are for ACh alone (*n* = 4–7, *N* = 1) and red open circles when ACh was co-applied with DMA and DEA (same cells than the control curve). All data were normalized to the maximal *I*_ACh_ elicited by ACh alone and fitted to Eq. (3). Continuous black and red lines are the fitted curves, labeled as Control_DEA+DMA_ and DEA+DMA, respectively. Added to this plot are the values we reported for the dose-response curves of nAChRs activated by ACh either alone (gray symbols and discontinuous line; Control_Lid_) or in the presence of 70 μM lidocaine (orange circles and discontinuous line; Lid; data from Alberola-Die et al., 2011). Notice the similarities among both control curves and between DEA+DMA and lidocaine curves.

A further experimental evidence for allotopic interaction of DMA and DEA was attained by determining the nAChR pharmacological profile in the presence of both DMA and DEA. Thus, nAChR bearing oocytes were challenged with different ACh concentrations (1 μM–1 mM) in the presence of 1 mM DMA and 30 μM DEA, which are the concentrations corresponding to their *IC*_30_ for nAChR blockade (see **Figure 1** and Alberola-Die et al., 2016, respectively). Then, if DMA and DEA interact with nAChRs at different loci, it should be expected roughly a 50% decrease in the *I*_ACh_ when applied together at these doses. Noticeably, the dose-response curve obtained in the presence of DMA and DEA fairly well-matched the pharmacological profile of nAChRs in the presence of the whole lidocaine molecule at its *IC*_50_ (**Figure 9**). Actually, we found non-significant differences between the values found for the dose-response curve in the presence of DEA and DMA and those previously attained in the presence of lidocaine (*p* > 0.05, *t*-test; Alberola-Die et al., 2011). Furthermore, the estimated *EC*_50_ value (65 μM) of nAChRs in the presence of DEA and DMA was within the confidence interval of the *EC*_50_ in the presence of lidocaine (64–147 μM). Therefore, as it could be expected from their differences in molecular structure, DEA and DMA act by different mechanisms and bind to different sites on nAChRs.

### Virtual Docking Assays

**FIGURE 10:**
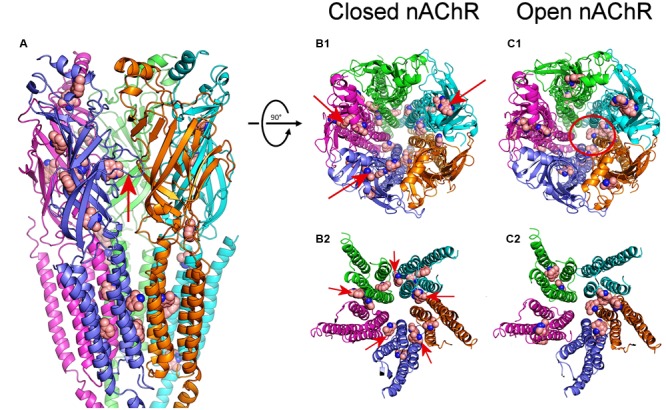
**Modeling of DMA binding to nAChR EC- and TM-domains in the open and closed states. (A)** Lateral view, in the membrane plane (top corresponding to the EC side) of nAChR, in the closed state, with bound DMA molecules. Subunits are colored for this and following panels as follows: α_1_ (blue), α_2_ (cyan), β (magenta), γ (orange), and δ (green). DMA molecules are colored brown and represented as van der Waals spheres. Notice that DMA binds both at the EC and TM domains. The red arrow indicates the orthosteric binding site at the α_1_-γ interface. **(B_1,_C_1_)** Top view (from the synaptic cleft) of nAChR structures in the closed **(B_1_)** and open **(C_1_)** states with bound DMA molecules. Note that, when closed, at the EC domain, DMA binds to intrasubunit loci (arrows in **B_1_**), mainly located on α_1_, α_2_ and β subunits, whereas at the TM domain DMA preferentially interact with residues located at intersubunit crevices. Also note that DMA binds within the channel pore only on nAChRs in the open state (red circle in **C_1_**). **(B_2,_C_2_)** Expanded top view of nAChR TM domains in the closed **(B_2_)** and open **(C_2_)** states with bound DMA. Note that whereas in the closed state DMA binds at all intersubunit assemblies (arrows in **B_2_**), in the open state these binding sites were less favored.

We have explored the interactions between DMA and the nAChR, using as template the full structure of *Torpedo* nAChR in both closed and open conformations (see Materials and Methods). We carried out 500 runs for DMA-nAChR interactions for both the closed and the open states. For the closed state, we found 60 clusters of interaction sites that differ in less than 5 Å of root-mean-square-deviation. DMA clusters on the nAChR were mainly located at the TM (52%) and EC (46%) domains, with only 1 cluster sited at the intracellular (IC) domain, sited adjacent to the TM region (**Figure 10A**). On the TM domain, DMA interacted both at intrasubunit crevices and at intersubunit interfaces, being these latter ones the more numerous, involving each single pair of nAChR subunits (**Figures 10B_1_,B_2_**). Interestingly, we found discernable changes on DMA binding to the TM domains in the closed and open states. Thus, in closed nAChRs DMA interacted preferentially with residues located at intra- and intersubunit spots but not into the channel pore (**Figures 10B_1_,B_2_**), whereas in the open state the hotspots for DMA were less numerous at intersubunit crevices (compare panels B_2,_C_2_ of **Figure 10**) and some appeared inside the channel pore (**Figure 10C_1_**, red circle). Noticeably, DMA binding sites on nAChR at the TM domain follow a pattern similar to that found for the entire lidocaine molecule on this receptor (see Supplementary Figure S1; Alberola-Die et al., 2016). At the EC domain, DMA bound at several intrasubunit crevices, mainly located on α_1_, α_2_, and β subunits (**Figure 10A**) and at the interface of α_1_-γ, γ-α_2_, δ-β, and β-α_1_ subunits. DMA was not found occupying the orthosteric sites, though there was a hotspot for DMA at the α_1_-γ interface relatively close to the ligand-binding site (**Figure 10A**).

## Discussion

We have studied the effect of DMA, which resembles the lipophilic aromatic ring of lidocaine, on muscle-type nAChRs, in order to unravel the structural determinants of the multiple inhibitory actions that lidocaine has on this receptor. As DEA (an analog of lidocaine’s hydrophilic moiety), DMA has inhibitory actions on nAChRs, but the two molecules differ in blocking potency, mechanisms of inhibition and binding sites on this receptor.

The blocking potency for DMA was in the millimolar range (**Figure 1**), which is far greater than the *IC*_50_ values found for either lidocaine or DEA (roughly 70 μM; Alberola-Die et al., 2011, 2016). Since DMA has a low pK_a_ (3.95; Gómez et al., 1972), most DMA molecules are unprotonated at physiological pH. In our recording solution (pH 7.0), the concentration of the uncharged form of lidocaine is roughly one-10th of the total species of lidocaine and, therefore, the DMA blocking potency would be roughly two orders of magnitude lower than that of neutral lidocaine. In consonance with this, using molecular properties of LAs as predictors of their affinity for nAChRs, Pagán et al. (2007) found that molecular weight, molecular volume, surface area and LogP (partition coefficient of the uncharged form between octanol/aqueous phases) of the hydrophilic portion of amide LAs (as lidocaine) correlated better with its *IC*_50_ than does the hydrophobic portion. Similarly, phenol, which also resembles the aromatic tail of lidocaine, caused the slow block of cardiac sodium channels seen with lidocaine, but its blocking potency was an order of magnitude lower (Zamponi and French, 1993). By contrast, 2,6-dimethylphenol, which has a molecular structure more analogous to DMA than just phenol, blocked either neuronal or skeletal-muscle sodium channels with a potency similar to that shown by the complete lidocaine molecule (Haeseler et al., 2002). Noteworthy, besides blocking voltage-gated sodium channels, 2,6-dimethylphenol potentiates and/or caused direct activation of GABA-A receptors (Krasowski et al., 2001; Mohammadi et al., 2001), which is opposite to the inhibitory effect that the whole lidocaine molecule has on this LGIC (Hara and Sata, 2007). In the case of DMA, at the concentrations used in this work, rather than enhancing GABA-A receptors decreased their activity (roughly 10% with 2 mM DMA) and, besides, slightly accelerated the GABA-A current decay (see Supplementary Figure S2).

The nAChR recovery from blockade by DMA was slower than that caused by either lidocaine or DEA (Alberola-Die et al., 2011, 2016) and much slower than the recovery from inhibition by the quaternary-ammonium anticholinesterases BW284c51 or edrophonium (Olivera-Bravo et al., 2007). This is most likely because DMA acts deeply into the membrane (note in **Figure 10** that most nAChR hotspots for DMA binding were located at the TM domain in the virtual docking assays), as it happens with neutral LAs (Pérez-Isidoro et al., 2014), which would increase its rinsing time. Actually, there are a variety of binding sites for hydrophobic molecules at the lipid-nAChR interface, mostly occupied by membrane phospholipids, but uncharged LAs, and likely DMA, might compete for these intramembranous binding sites (Mantipragada et al., 2003). In this sense, it should be pointed out that lidocaine, and likely other amphipathic molecules, might follow both hydrophobic and hydrophilic pathways through the membrane to reach their deep binding sites in the voltage-dependent sodium channels (Hille, 1977), and something similar would be expected for the nAChR.

2,6-Dimethylaniline caused a voltage-independent blockade of nAChRs (**Figure 3**), likely by acting outside the channel pore on resting nAChRs (see below). Nevertheless, some neutral LAs, as benzocaine, can block open nAChR channels (Ogden et al., 1981). Interestingly, we found prominent *I*_ACh_ rebounds after co-application of ACh and DMA, when DMA began to be rinsed out (**Figures 4** and **6**). This *I*_ACh_ rebound might arise if DMA binds with low affinity into the open channel pore, as it has been proposed for ACh (Legendre et al., 2000; Liu et al., 2008) and other fast channel blockers, as TMA or choline (Lape et al., 2009). Thus, when DMA concentration decreases by washout, the channel would be unplugged, at a time when there is yet enough ACh to keep some nAChRs open. The DMA low-affinity binding would explain why *I*_ACh_ rebound was only elicited when DMA concentration was over 500 μM. The larger *I*_ACh_ rebound found when the cell was challenged with a high ACh concentration could be due to the presence of a larger remanent ACh concentration during the washout, which is required for nAChR activation. Accordingly, virtual docking assays show a hotspot for DMA binding inside the pore in the open nAChR conformation (**Figure 10C_1_**). Alternatively, *I*_ACh_ rebound could be due to low affinity binding of DMA to the nAChR TM domain at intersubunit crevices (**Figure 10B_2_**), provided this binding precludes channel opening by ACh. As shown in Supplementary Figure S1, the pattern of DMA binding to intersubunit cavities in the nAChR is very similar to that found for the entire lidocaine molecule (Supplementary Figure S1; Alberola-Die et al., 2016) and also for the general anesthetics propofol (Supplementary Figure S1; Ghosh et al., 2013) and isoflurane (not shown; Brannigan et al., 2010) on the homologous GLIC receptor, in the resting state. Interestingly, motions at intersubunit crevices elicited by propofol on GLIC receptors seem different to those associated with channel activation, which suggests that propofol stabilize this receptor in the closed state (Ghosh et al., 2013), and something similar might occur with DMA and lidocaine on nAChRs. If this is so, lowering DMA concentration by rinsing would remove this restriction for nAChR gating and thus *I*_ACh_ rebound would initiate; however, changes in DMA concentration at deep membrane loci would probably follow a time course too slow for keeping nAChR activated by the remaining ACh, which is required to initiate the *I*_ACh_ rebound.

So far, the amine group of the lidocaine molecule (or its analog DEA) has been proposed as the single molecular determinant for the open-channel blockade of nAChRs (Alberola-Die et al., 2011, 2016). By contrast, the aromatic ring of lidocaine (or its analog DMA) arises as the structural determinant for the enhancement of the *I*_ACh_ decay elicited by lidocaine, since this effect could neither be elicited by DEA, even at concentrations threefold the *IC*_50_ (Alberola-Die et al., 2016), nor by low concentrations of lidocaine (Alberola-Die et al., 2011). The *I*_ACh_ decay enhancement by DMA can be explained by two, not exclusive, mechanisms: (i) by slow open-channel blockade of nAChRs, which would account for the *I*_ACh_ rebound at the beginning of DMA rinsing. However, the fact that for the same DMA concentration the *I*_ACh_ decay time course was markedly affected by ACh dose (**Figure 6**) excludes this mechanism as the only one responsible for this effect; (ii) by hastening nAChR desensitization, which would better explain the differences in the rates of *I*_ACh_ decay found for different ACh concentrations in the presence of the same dose of DMA. If this is so, the enhancement of nAChR desensitization can be evoked by direct ACh and DMA co-application, without requiring DMA preincubation, in contrast with the enhancement of muscle nAChRs desensitization by adiphenine, which required 2 min preincubation with this LA to reach the maximum effect (Spitzmaul et al., 2009).

When DMA was pre-applied to the oocyte before its co-application with ACh, nAChR blockade markedly increased, mainly at high ACh doses, which strongly suggests that DMA blocked closed nAChRs. Furthermore, in competition assays, DMA decreased the maximum ACh-elicited response, as it would be expected for a non-competitive blocker, but also shifted significantly to the right the dose-response curve (**Figure 7**). This apparent competitive effect of DMA could be explained by blockade of resting nAChRs, since it precludes their activation by the agonist, i.e., it would decrease the total number of nAChRs available for activation. In concordance with this, virtual docking assays indicated that DMA binds outside the channel pore in the resting nAChR, mainly at intersubunit crevices of TM and EC domains (**Figures 10A,B_1_,B_2_**).

Whereas DMA binding sites at TM segments of nAChRs where rather similar to those found for lidocaine (Supplementary Figure S1), they were strikingly different to those reported for DEA (see Alberola-Die et al., 2016), indicating that DMA and DEA caused specific actions on nAChRs by acting at different loci. This result was confirmed by analyzing the additive inhibitory actions caused by co-application of ACh with DEA and DMA as compared with co-application of ACh with either DEA or DMA. As it would be expected from our docking assays, the increase in the percentage of *I*_ACh_ inhibition was very close to the value estimated from the allotopic model of interaction of two blockers on an ion channel (Jarvis and Thompson, 2013). Therefore, DEA and DMA can simultaneously occupy their specific binding sites on this receptor, eliciting each one of these molecules selective effects on nAChRs, and when DEA and DMA are acting together they reproduce most of the inhibitory actions elicited by the whole lidocaine molecule, including the pharmacological profile of nAChRs exposed to lidocaine (see **Figure 9**). Thus, these results allow to explain why when lidocaine is applied at low doses (below *IC*_50_) it matched most DEA blocking actions, but at higher doses, it showed additional inhibitory effects, mimicking some DMA actions.

## Conclusion

These results indicate that many amphipathic molecules, including most LAs, might exert a complex modulating action on nAChRs by simultaneously acting, with different affinities, at distinct and even distant binding sites on this receptor and, most likely, this is also suitable for other LGICs.

## Author Contributions

All signing authors have contributed substantially to the conception of this work and to the acquisition, analysis and interpretation of the data presented. Besides, all of them have participated in drafting and revising the submitted manuscript and have approved the version submitted for publication.

## Conflict of Interest Statement

The authors declare that the research was conducted in the absence of any commercial or financial relationships that could be construed as a potential conflict of interest.

## Footnotes

1 http://www.ncbi.nlm.nih.gov/pccompound

2 http://www.pymol.org/

## Abbreviations

ACh: acetylcholine
ANR: normal Ringer solution with atropine
BW284c51: 1,5-bis(4-allyldimethylammoniumphenyl)pentan-3-one dibromide
DEA: diethylamine
DMA: 2,6-dimethylaniline
DMSO: dimethyl sulfoxide
EC: extracellular
*I*_ACh_: ACh-elicited current
IC: intracellular
LA: local anesthetic
LGIC: ligand-gated ion channel
MS-222: ethyl 3-aminobenzoate methanesulfonate
*n*: number of oocytes
*N*: number of oocyte-donor frogs
nAChR: nicotinic acetylcholine receptor
NR: normal Ringer solution
QX-222: 2-(trimethylammonio)-*N*-(2,6-dimethylphenyl) acetamide chloride
QX-314: 2-(triethylammonio)-*N*-(2,6-dimethylphenyl) acetamide bromide
TEA: tetraethylammonium
TM: transmembrane spanning-segment
TMA: tetramethylammonium
