# Supplementary material for: Muscle-Type Nicotinic Receptor Modulation by 2,6-Dimethylaniline, a Molecule Resembling the Hydrophobic Moiety of Lidocaine
Source: Front Mol Neurosci. 2016 Nov 24;9:127. doi: 10.3389/fnmol.2016.00127 (PMC5121239; doi:10.3389/fnmol.2016.00127)
Supplement: Supplementary file 1 [file Presentation_1.pdf]

Supplementary Material

Muscle-type nicotinic receptor modulation by 2,6-dimethylaniline, a molecule resembling the hydrophobic moiety of lidocaine

Armando Alberola-Die[1], Gregorio Fernández-Ballester[2], José Manuel González-Ros[2], Isabel Ivorra[1] and Andrés Morales[1,*]

[1]División de Fisiología, Departamento de Fisiología, Genética y Microbiología, Universidad de Alicante, Apdo. 99, E-03080 Alicante, Spain.

[2]Instituto de Biología Molecular y Celular, Universidad Miguel Hernández, Elche, E-03202, Alicante, Spain

*** Correspondence:**

Andrés Morales

andres.morales@ua.es

Phone: 34-965903949

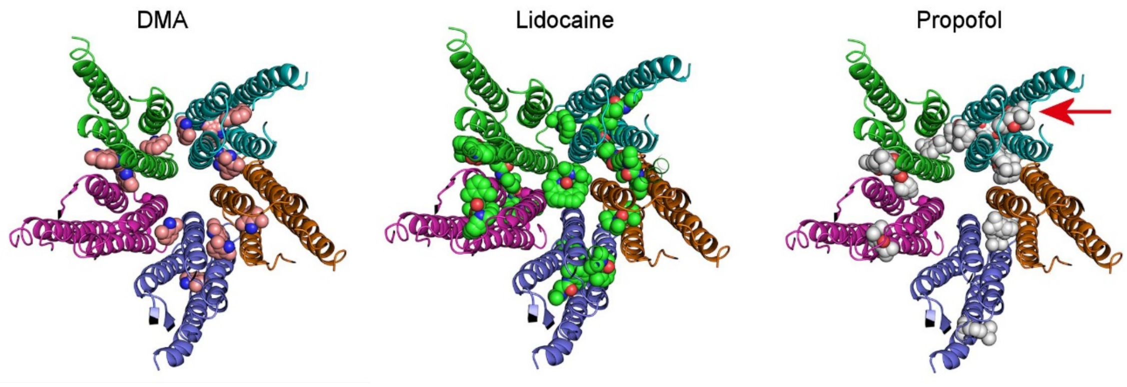

Supplementary Figure S1. *Virtual docking assays comparing DMA binding sites at the nAChR transmembrane (TM) domain in the closed state to those of lidocaine and propofol.* nAChR subunits are coloured as follows: α (blue and cyan), β (magenta), γ (orange) and δ (green). All ligands are represented in van der Waals spheres, being DMA molecules colored in brown, lidocaine in green and propofol in grey. (***Left***) Top view of the TM domain showing DMA binding sites, mainly located at intersubunit crevices. (***Middle***) Lidocaine binding on the TM domain of nAChR matched fairly well the DMA binding sites at intersubunit crevices and, besides, interacted within the channel pore. **(*Right*)** A similar view of the nAChR showing the propofol binding sites. Note the similarities among the binding sites at intersubunit crevices for DMA, lidocaine and propofol. As DMA, propofol does not bind into the channel pore but has an intrasubunit binding site at the α_2 chain (red arrow), which is common to both DMA and lidocaine; furthermore, this intrasubunit binding site is analog to that reported for propofol binding on GLIC receptors (PDB code: 3P50; Gosh *et al.*, 2013; *doi: 10.1074/jbc.M113.464040*).

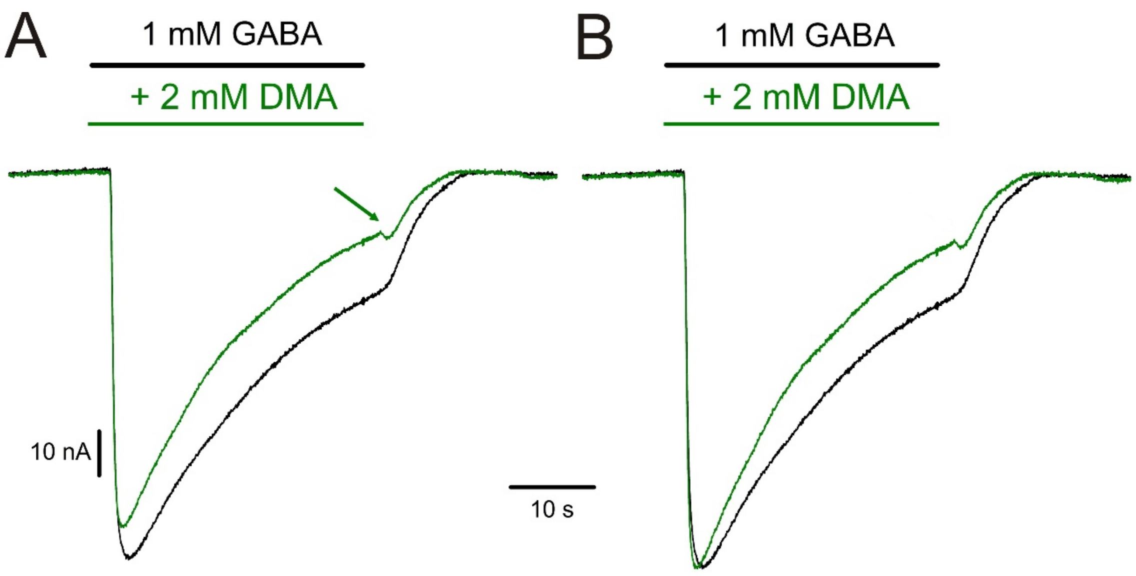

Supplementary Figure S2. *Effect of DMA on GABA-elicited currents.* $GABA_A$ receptors ($GABA_ARs$) from rat brain synaptosomal-enriched membranes, obtained as previously described by Alberola-Die *et al*., 2016 (Front. Mol. Neurosci. 9:12; *doi: 10.3389/fnmol.2016.00012*), were microtransplanted to the *Xenopus* oocyte membrane. **(A)** GABA-elicited currents (I_{GABA}) were recorded, at a holding potential of -60 mV, by superfusing the cell with 1 mM GABA either alone (control, black recording) or together with 2 mM DMA (green recording). Notice that 2 mM DMA caused a small I_{GABA}-peak inhibition (12 ± 6%, n=4) and slightly enhanced I_{GABA} decay, as better evidenced in panel **(B)**, where both recordings were normalized to the same amplitude. Interestingly, rebound currents (arrow in panel A) were observed during DMA washout, suggesting that this molecule binds into the channel pore of $GABA_ARs$.
